# Effect of Soluble ICAM-1 on a Sjögren's Syndrome-like Phenotype in NOD Mice Is Disease Stage Dependent

**DOI:** 10.1371/journal.pone.0019962

**Published:** 2011-05-12

**Authors:** Nienke Roescher, Jelle L. Vosters, Hongen Yin, Gabor G. Illei, Paul P. Tak, John A. Chiorini

**Affiliations:** 1 Division of Clinical Immunology and Rheumatology, Academic Medical Center, University of Amsterdam, Amsterdam, The Netherlands; 2 Molecular Physiology and Therapeutics Branch, National Institute of Dental and Craniofacial Research, National Institutes of Health, Bethesda, Maryland, United States of America; University of Southern California, United States of America

## Abstract

**Introduction:**

Intercellular adhesion molecule-1 (ICAM-1) is involved in migration and co-stimulation of T and B cells. Membrane bound ICAM-1 is over expressed in the salivary glands (SG) of Sjögren's syndrome (SS) patients and has therefore been proposed as a potential therapeutic target. To test the utility of ICAM-1 as a therapeutic target, we used local gene therapy in Non Obese Diabetic (NOD) mice to express soluble (s)ICAM-1 to compete with membrane bound ICAM-1 for binding with its receptor. Therapy was given prior to and just after the influx of immune cells into the SG.

**Methods:**

A recombinant serotype 2 adeno associated virus (rAAV2) encoding ICAM-1/Fc was constructed and its efficacy tested in the female NOD mice after retrograde instillation in SG at eight (early treatment) and ten (late treatment) weeks of age. SG inflammation was evaluated by focus score and immunohistochemical quantification of infiltrating cell types. Serum and SG tissue were analyzed for immunoglobulins (Ig).

**Results:**

Early treatment with ICAM-1/Fc resulted in decreased average number of inflammatory foci without changes in T and B cell composition. In contrast, late treated mice did not show any change in focus scores, but immunohistochemical staining showed an increase in the overall number of CD4+ and CD8+ T cells. Moreover, early treated mice showed decreased IgM within the SGs, whereas late treated mice had increased IgM levels, and on average higher IgG and IgA.

**Conclusions:**

Blocking the ICAM-1/LFA-1 interaction with sICAM-1/Fc may result in worsening of a SS like phenotype when infiltrates have already formed within the SG. As a treatment for human SS, caution should be taken targeting the ICAM-1 axis since most patients are diagnosed when inflammation is clearly present within the SG.

## Introduction

Intercellular adhesion molecule-1 (ICAM-1) binds to lymphocyte function-associated antigen-1 (LFA-1) and macrophage 1 antigen (Mac-1) on immune cells, and is involved in adhesion and migration of leucocytes in an inflammatory environment. ICAM-1 also plays an important role in the co-stimulatory pathway involved in T cell activation and clonal expansion [1], and T cell dependent B cell activation [2]. ICAM-1 is upregulated in endothelial cells, lymphocytes, fibroblasts, and ductal epithelium of salivary glands (SG) from Sjögren's syndrome (SS) patients [3], [4], [5], [6]. SS is a systemic autoimmune disorder affecting secretory tissue, including the lachrymal and salivary glands, resulting in keratoconjunctivitis sicca and xerostomia. One of the pathological hallmarks of the disease is the focal infiltration of mononuclear cells into these secretory glands.

Currently, there is no effective treatment for SS. Since ICAM-1 is consistently found to be upregulated in SS, it has been suggested that targeting ICAM-1 and the interaction with its ligands may positively affect the disease outcome [7], [8]. In previous studies, blocking ICAM-1 interaction by systemic administration of sICAM-1, has proven to be an effective therapy for autoimmune diabetes in the Non Obese Diabetic (NOD) mouse. Intraperitoneal (ip) injection with sICAM-1 before the clinical onset of disease in NOD mice, resulted in decreased monocytic infiltration into the pancreas, reduced Th1 cytokines levels and a lower diabetes incidence [9]. Another study showed that treatment of NOD mice with sICAM-1 after the onset of diabetes resulted in long-term remission of diabetes in >50% of treated mice. Interestingly, remission was not accompanied by decreased diabetogenic T cells and did not result in overall immunosuppression, suggesting induction of tolerance by sICAM-1 [10].

Independent of diabetes [11], the NOD mice also spontaneously develop a complex of features that resembles SS in humans [11]. These mice spontaneously develop SG focal infiltrates, mainly consisting of B and T cells, and within the inflamed SG, membrane bound endothelial and epithelial ICAM-1 and LFA-1 are upregulated [12]. These characteristics make the NOD mouse a reasonable model to study the potential therapeutic effect of ICAM-1 interference on the development of SS.

Since ICAM-1 plays a role in the migration of immune cells into tissue, we tested the effect of sICAM-1 overexpression and secretion by ductal cells in SG of NOD mice, before (early treatment) and after (late treatment) the influx of inflammatory cells, to see whether we can intervene with the formation of the first focal infiltrates. The ductal cells are thought to play a crucial role in the pathogenesis of SS [13] since focal infiltrates in SS are mainly found surrounding the ductal epithelial cells. Moreover, ductal cells produce high levels of pro-inflammatory cytokines and can act as nonprofessional antigen presenting cells [14], making these cells an attractive target. In this study, we investigated whether sustained expression of sICAM-1 by ductal epithelial cells via local gene therapy in the SG of NOD mice could affect the development of SS-like clinical and immunological symptoms.

## Results

### Natural expression of ICAM-1 in the SGs

**Figure 1:**
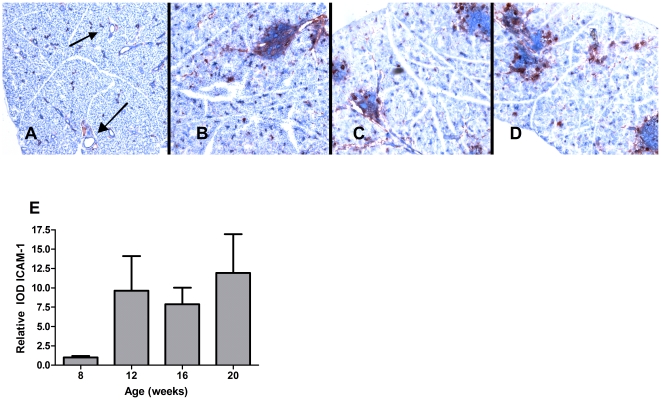
Natural ICAM-1 expression in NOD SGs. Frozen sections from 8 (N = 5), 12 (N = 5), 16 (N = 4), and 20 (N = 5) week old non-treated NOD SGs were analyzed and quantified for natural ICAM-1 expression. Representative images (400× magnification) of ICAM-1 expression in NOD SGs are shown at 8 (**A**), 12 (**B**), 16 (**C**), and 20 (**D**) weeks of age. Arrows indicate ICAM-1 expression at ductal and endothelial epithelium. **E**) Quantification of ICAM-1 expression for all ages. Data shown are mean values +/− SD. The P-value was determined by the parametric Student's t-test.

ICAM-1 expression was determined in the SGs of NOD mice at the age of 8, 12, 16 and 20 weeks. Eight week old NOD mice do not have focal infiltrates, but already expressed ICAM-1 in the ductal and endothelial epithelium (Figure 1A). From 12 weeks to 20 weeks of age, when infiltrating lymphocytes are clearly present, ICAM-1 was still expressed within the epithelial and endothelial cells, but was also strongly expressed in the focal infiltrates (Figure 1B–D). Quantification of ICAM-1 showed a nearly 10-fold increase (p<0.005) of expression levels between 8 and 12 weeks of age, and stayed stable after 12 weeks (Figure 1E). This increase was based on infiltrating cells, since increase in expression of the ductal and endothelial cells did not change (data not shown).

### *In vitro* ICAM-1/Fc is secreted as a dimer and is biologically active

**Figure 2:**
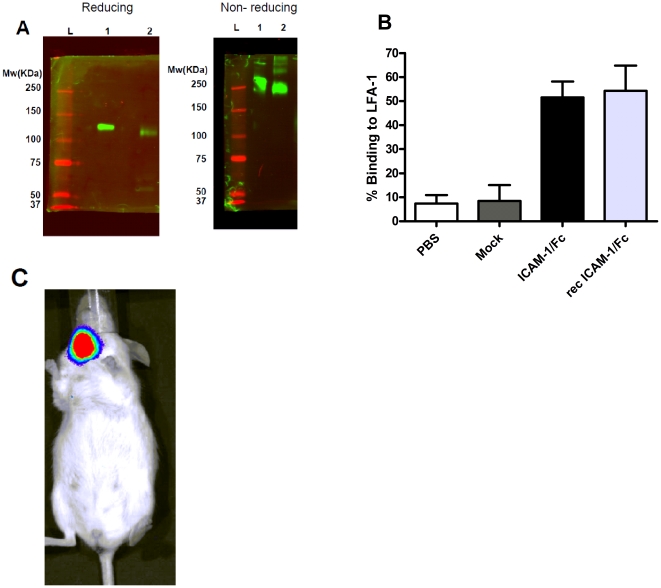
*In vitro* and *in vivo* expression and biological activity of ICAM-1/Fc. **A**) *In vitro* expression of ICAM-1/Fc was shown using a reducing and nonreducing SDS-PAGE gel. Under reducing conditions, ICAM-1/Fc migrated as a monomer with a molecular mass of approximately 120 kDa and under nonreducing conditions as a dimer with a molecular mass of approximately 240 kDa. Lane 1 is recombinant ICAM-1/Fc and lane 2 is ICAM-1/Fc-transfected supernatant. **B**) The biological activity of the ICAM-1/Fc was determined by adding stimulated and labeled T cell blastoma cells to plates coated with ICAM-1/Fc-transfected supernatant. The optical density between before and after washing the wells is expressed as percentage binding to LFA-1. Data shown are mean values +/− SD of 3 experiments. PBS = phosphate buffered saline coated wells, mock = mock-transfected supernatant coated cells. **C**) *In vivo* imaging of luciferase expression within the SGs. One representative mouse is shown.

ICAM-1/Fc was cloned into an AAV2 vector and the vector was tested for protein expression after transfection of human embryonic kidney (HEK 293) cells. Supernatant was harvested 48 hrs after transfection and a western blot was performed under reducing and nonreducing conditions. ICAM-1/Fc secreted in the supernatant migrated as expected as a dimer with a molecular mass of approximately 240 kDa under nonreducing conditions and as a monomer at 120 kDa under reducing conditions, similar to commercially available recombinant ICAM-1/Fc (Figure 2A). Supernatant was tested in an ELISA and 0.62 ug/ml of fusion protein was detected, which was within the expected range for AAV-plasmid expression (data not shown). An *in vitro* biological activity assay showed a 52% binding of activated lymphocytes, over expressing LFA-1, to wells coated with supernatant containing ICAM-1/Fc, similarly to the commercially available recombinant ICAM-1/Fc (Figure 2B).

### Confirmation of local transgene delivery and stable transduction of the SG *in vivo* after local administration

SGs of NOD mice of 8 or 10 weeks of age were cannulated with AAV2-vectors containing ICAM-1/Fc or LacZ together with a luciferase reporter vector. We used 1×10^11^ viral particles per SG since previous studies from our group have shown that the optimal dose for protein expression without causing a significant immune response lies between 1×10^10^ and 1×10^11^ particles per gland [15], [16]. Prior to sacrifice, mice were anesthetized and were given luciferin ip, after which luminescence was measured by an in vivo luminescence imager (IVIS) to ensure localized transfer of the vectors to the SGs. Figure 2C shows a representative mouse expressing luciferase in the SG, 10 weeks after a single administration. Vector delivery was also confirmed using quantitative polymerase chain reaction (qPCR) on total DNA extracted from treated SGs (data not shown). In agreement with previous studies by our group expression was localized to the salivary gland and not detected in other tissues of the animal (data not shown).

### Early treatment decreased overall inflammation of the SGs

**Figure 3:**
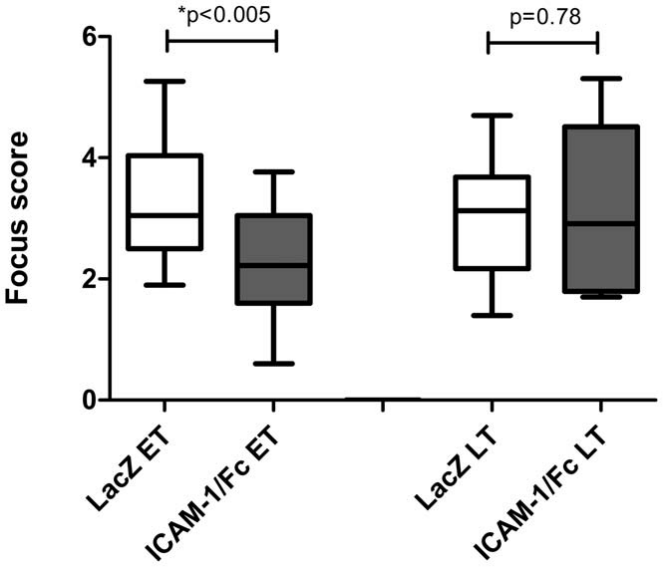
Decreased focus score in early treated mice. SGs from early treated mice (N = 16 for LacZ and N = 19 for ICAM-1/Fc) and late treated mice (N = 8 for LacZ and N = 9 for ICAM-1/Fc) were removed for histologic analysis to assess the overall inflammation. Data are shown as box and whisker plots with the mean and 5–95 percentile. The P-value was determined by the parametric Student's t-test. ET = early treatment, LT = late treatment.

To determine the effect of treatment with ICAM-1/Fc on inflammation of the SGs, NOD mice treated at 8 weeks (early treatment) or at 10 weeks (late treatment) of age were euthanized at 20 weeks and the focus score (FS) was determined. FS in early ICAM-1/Fc-treated mice were significantly lower compared with LacZ-treated mice (2.3±0.9 versus 3.3±1.0, p<0.005). In contrast, late treatment did not affect the FS (3.2±1.4 versus 3.0±1.1, p = 0.78) (Figure 3).

### Late ICAM-1/Fc treatment increased CD4+ and CD8+ T cells in the SG

**Figure 4:**
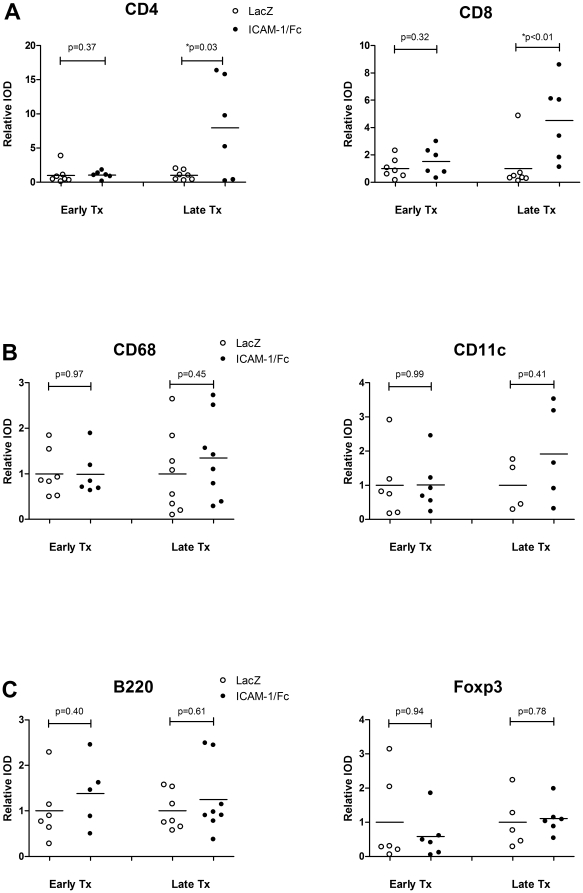
Increased relative IOD of CD4+ and CD8+ T cells in late treated mice. SGs from early and late treated mice with an average of 7 mice per group were quantified for CD4+ T cells, CD8+ T cells (**A**), CD68+ macrophages, CD11c+ dendritic cells (**B**), and B220+ B cells and Foxp3+ regulatory T cells (**C**). Data shown are the mean relative IOD. The P-value was determined by the parametric Student's t-test or the non-parametric Wilcoxon's ranksum test depending on the Gaussian distribution for each staining.

To identify the cell types affected by the ICAM-1/Fc treatment, we performed immunohistochemistry (IHC) analysis and quantification of the foci of SG sections from early and late treated mice. Despite the reduced number of foci, no specific cell type was found to be reduced within these foci (Figure 4A). In contrast, late treated mice showed a significant number of mice with an increased relative integrated optical density (IOD) of positive CD4+ (p = 0.03) and CD8+ T cell staining (p<0.01) (Figure 4A). No differences in the relative IOD of macrophages (CD68), dendritic cells (CD11c), B cells (B220+) and Treg (Foxp3+) were detected in both early (p = 0.97, p = 0.99, p = 0.40, and p = 0.94 respectively) and late (p = 0.45, p = 0.41, p = 0.61, and p = 0.78 respectively) treated mice (Figure 4B–C).

### ICAM-1/Fc can affect local and systemic immunoglobulin levels and is disease stage dependent

**Figure 5:**
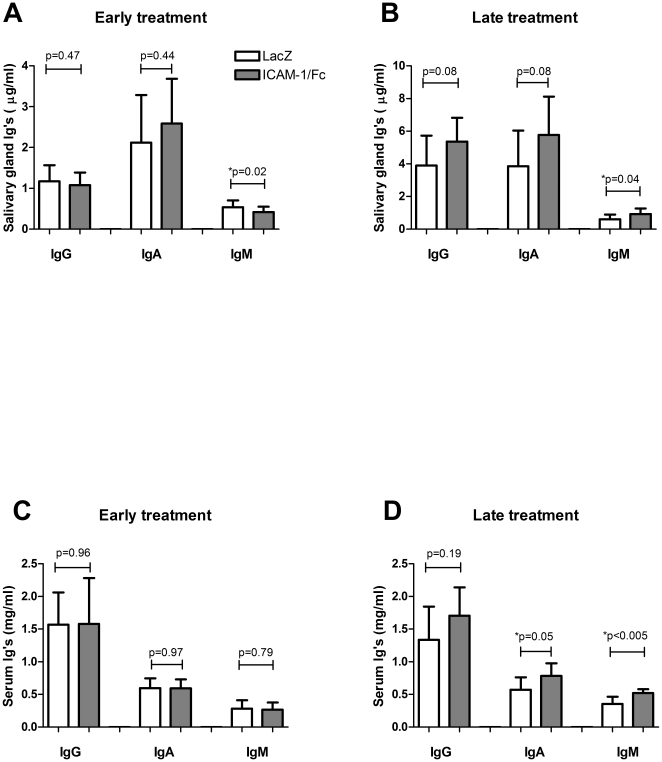
The timing of ICAM-1/Fc delivery affects local and systemic Ig levels. SG homogenates (**A–B**) and serum (**C–D**) from early (average of N = 8 per group) and late (average of N = 18 per group) treated mice were analyzed for IgG, IgA and IgM. Data shown are mean +/− SD. The P-value was determined by the parametric Student's t-test or the non-parametric Wilcoxon's ranksum test depending on the Gaussian distribution for each Ig.

ICAM-1 is involved in T cell activation and T cell dependent B cell activation. Therefore, we studied whether interrupting the ICAM-1/LFA-1 interaction would alter local and systemic immunoglobulin (Ig) levels. Early treatment showed decreased levels of IgM in the ICAM-1/Fc treated SGs compared to LacZ controls (0.42±0.13 µg/ml versus 0.54±0.17 µg/ml, p = 0.02); IgG (p = 0.47) and IgA (p = 0.44) levels were unchanged (Figure 5A). In contrast, late treatment with ICAM-1/Fc resulted in increased levels of IgM (0.93±0.35 µg/ml versus 0.60±0.29 µg/ml, p = 0.04) in the SGs, and tended to increase local IgG (5.36±1.47 µg/ml versus 3.90±1.82 µg/ml, p = 0.08) and IgA levels (5.78±2.34 µg/ml versus 3.85±2.19 µg/ml, p = 0.08) (Figure 5B). Ig serum levels in the early treated mice were unaltered (Figure 5C). In contrast, late ICAM-1/Fc treated mice showed increased serum levels of IgM (0.52±0.06 mg/ml versus 0.36±0.11 mg/ml, p<0.005), and IgA (0.78±0.19 mg/ml versus 0.57±0.19, p = 0.05), and tended to increase serum IgG levels (1.71±0.44 mg/ml versus 1.34±0.51 mg/ml, p = 0.19) (Figure 5D).

### Salivary flow was unaltered after ICAM-1/Fc delivery

NOD mice in our facility start to develop decreased salivary flow from 16 weeks of age (Roescher et al, unpublished data). To study whether the reduced SG inflammation after ICAM-1/Fc treatment affected the salivary flow, we measured stimulated salivary flow at the end of study (age 20 weeks). Despite the observed reduction in focus score, stimulated salivary flow was not improved in early treated mice. In addition, to make sure we did not miss the window of effect of sICAM-1, we also tested early treated mice at 16 weeks of age and also in this group no difference in salivary flow was observed. The late treatment group also did not show any difference in salivary flow compared with controls despite the increase observed in SG infiltrating T cells (data not shown).

## Discussion

ICAM-1 is upregulated in many inflammatory diseases. It functions as an adhesion molecule and plays a role in the interaction and reciprocal activation of B and T cells by binding to LFA-1 and Mac-1. Targeting the interaction between ICAM-1 and LFA-1 as a treatment for (auto)immunity has been the subject in a number of studies (reviewed in [17]), and was shown to have beneficial effects in animal models for autoimmune diseases [9], [10]. ICAM-1 and LFA-1 are upregulated in SG of SS patients [3], [4], [5], [6] suggesting an important role for these molecules in this disease. This is further supported by the systemic administration of antibodies to LFA-1 in NOD mice, which resulted in inhibition of infiltration of the lachrymal glands [18]. Moreover, *in vitro* stimulation of human salivary gland (HSG) ductal cells with interferon (IFN)-γ gamma, a cytokine central to SS [19], in the presence or absence of tumor necrosis factor (TNF), increased the expression of ICAM-1 on the cell surface [20]. It has therefore been speculated that interfering with the ICAM-1/LFA-1 interaction may improve the clinical outcome in SS. To study this, sICAM-1/Fc was expressed in the SG of NOD mice in an initiation phase and in a more advanced stage of a SS-like disease and the effect on inflammatory parameters was analyzed.

NOD mice start to form SG infiltrates from 8 weeks of age. At this age, prior to the development of infiltrates, mice clearly expressed ICAM-1 in the ductal epithelium and endothelium. Four weeks later the overall ICAM-1 expression was increased 10 fold, based on the expression of infiltrating cells. These data confirm that endothelium and epithelium are already activated prior the influx of lymphocytes and suggest that ICAM-1 signaling is important in an early phase of the pathogenesis of SS in these mice. Based on these results and on existing literature indicating that timing of ICAM-1 interference can determine the outcome [21], we hypothesized that treatment with ICAM-1/Fc at an initiation phase of the disease may differ from and be more effective than treatment later in the disease course. Therefore we treated 8 week old mice, just before inflammatory cells are entering the SG, and at 10 weeks at an accelerated phase of the disease when distinct foci have formed in the majority of mice (N. Roescher et al, manuscript in preparation).

Mice were cannulated with a non-replicating AAV type 2 vector, known to stably transduce ductal epithelial cells in the SG [22]. Indeed, at the age of 20 weeks, vector DNA of ICAM-1/Fc was still detected in the SG indicating stable transduction. Moreover, *in vivo* imaging of luciferase activity, which was cotransduced during administration of ICAM-1/Fc, showed localized expression in the SG. No luciferase activity was detected in the liver or in any other organ, indicating no systemic spread (data not shown).

Inflammation of the SG was reduced in the early treated mice, although no reduction of any specific cell type was found within the foci, suggesting a general block on the influx of cells into the SG. Interestingly, although the absolute FS was unchanged in the late treatment group, the relative IOD of CD8+ cells and CD4+, but not of Treg (Foxp3+) cells was increased compared with the control mice. We also noted a ∼2 fold increase in the average size of the foci (data not shown), suggesting a specific recruitment to or local expansion of effector T cells in the SG foci. These data is consistent with previously reported T cell proliferation and IL-2 secretion after incubation with sICAM-1 *in vitro*, indicating direct or indirect T cell activation by sICAM-1 [10]. In addition, the contrasting effects observed in the different timing of treatment indicate a shift in the role for therapeutic sICAM-1/Fc over the course of disease progression. Although we did not study this in detail, this notion is also supported by literature. Recently, anti-ICAM-1 antibodies have been shown to positively affect the clinical course of experimental autoimmune encephalitis (EAE) when given in an early phase of the disease, whereas treatment given in a later phase, worsened the clinical score of the treated mice [21]. Based on these data and on the early expression of endogenous ICAM-1 in the SG of mice, it would be interesting to study sICAM-1/Fc delivery at an earlier age than 8 weeks. However, the SG of NOD mice are not fully developed until the age of 8 weeks and hence the orifices of the main ducts of the submandibular gland draining into the oral cavity are very small making it technically not feasible to cannulate the glands before this age.

In parallel to our animal study, a clinical study was undertaken at the National Institutes of Health (NIH) in patients with SS using a monoclonal antibody to the CD11a subunit of LFA-1 (efalizumab), a ligand of ICAM-1. Unexpectedly, this treatment increased inflammation in minor salivary glands and development of de novo autoantibodies was detected in some patients [23]. Although this therapeutic approach is different from the approach we present in this paper (e.g. the use of a monoclonal antibody versus a soluble molecule), these results may further indicate that blocking the ICAM-1/LFA-1 interaction may cause adverse effects once inflammation is established.

Many SS patients have high levels of circulating immunoglobulins, indicating B cell activation. Since ICAM-1 is involved in T cell dependent B cell stimulation, directly or via the production of IL-2 by activated T cells, we measured the effect of sICAM-1 on local immunoglobulin production. Immunoglobulin levels in the SG were differentially affected based on the timing of sICAM-1/Fc expression, with the most striking differences observed in IgM levels, suggesting again a dichotomous role for sICAM-1 in the course of murine SS. Little is known about the direct role of ICAM-1 on immunoglobulin synthesis, but the effects observed could be the result of direct B cell stimulation or indirect stimulation via activation of T cells by sICAM-1/Fc. Additional research is required to elucidate the exact mechanism.

Stimulated salivary flow was unchanged despite reduced inflammation in the early treatment group, indicating that infiltration of the SG by lymphocytes in the early treated group was not sufficiently suppressed. On the other hand, salivary flow was also unchanged in the late treatment group, despite the increase of CD4+ and CD8+ T cells in the SG, suggesting that T cells may not play a direct role in SG dysfunction of the inflamed glands. Most importantly, these data imply that the loss of salivary flow in SS is the end result of a complicated pathological process that is poorly understood.

In summary, the expression of sICAM-1 in the SG of NOD mice led to a modest decrease in autoimmune sialadenitis when treatment was given at an early stage of the disease. However, late treatment increased the number of CD4+ and CD8+ T cells in the SG, and immunoglobulin levels in the SG and serum, indicating a dichotomous role for sICAM-1 on the pathogenesis of murine SS depending on disease stage. These data also indicate that caution must be taken in treating human SS with therapies targeting the ICAM-1/LFA-1 interaction, since it is likely that most patients are diagnosed and request treatment in a more progressed stage of the disease.

## Materials and Methods

### Vector design and *in vitro* expression

The plasmid for mouse ICAM-1 coupled to the Fc-part of mouse immunoglobulin G1 (IgG1) was kindly provided by Dr. Lemarchand (Université René Descartes, France). We previously reported the construction of recombinant (r)AAV-LacZ encoding β-galactosidase [24]. The plasmid for rAAV-luciferase was a kind gift of Dr. Mizukami (Jichi Medical School, Japan). Each gene was cloned into an rAAV plasmid containing a cytomegalovirus (CMV) promoter and the inverted terminal repeat (ITR) sequences for AAV2.

The resulting plasmid (pAAV2-CMV-mICAM-1-mIgG1) was transfected into HEK 293 cells and protein secretion into the supernatant was quantified by an ELISA kit for mouse ICAM-1 (R&D systems, Minneapolis, MN). The size of the fusion protein (ICAM-1/Fc) was confirmed by western blotting of the supernatant after transfection under reduced and nonreduced conditions using a 10% SDS gel, rat anti-mouse ICAM-1 (R&D systems) as a primary antibody and a labeled (IRDye 800 CW) goat anti-rat IgG (Li-Cor, Lincoln, NE) as a secondary antibody. A recombinant mouse ICAM-1/Fc chimera (R&D systems) was used as a positive control.

### *In vitro* biological activity

The biological activity of the expressed soluble ICAM-1/Fc was tested *in vitro* for the ability to bind LFA-1. A flat bottom 96-well plate was coated overnight (O/N) at 4°C with supernatant containing ICAM-1/Fc or recombinant ICAM-1/Fc (R&D systems) as positive control. Human T cell blastoma cells (HSB2, ATCC, Manassas, VA) were stimulated with phorbol 12-myristate 13-acetate (PMA, Sigma-Aldrich, St. Louis, MO) at 50 nM final concentration for 30 minutes (min) at 37°C to increase LFA-1 expression on the membrane. Cells were labeled with fluorescent calcein-AM solution (Invitrogen, Carlsbad, CA) at 25 µM final concentration for 30 min at 37°C. Stimulated and labeled HSB2 cells (5×10^4^ cells/well) were incubated for 1.5 hr at 37°C in the pre-coated wells. The plate was read before and after washing at 485–538 nm in a Spectramax M2 plate reader (Molecular Devices Corporation, Sunnyvale, CA). The difference in optical density between before and after washing was expressed as percentage adhesion.

### Mice

NOD mice (001976 NOD/ShiLtJ, Jackson, Bar Harbor, ME) were ordered at 6 weeks of age and were kept in pathogen-free conditions. All procedures involving animals were performed in compliance with the National Institutes of Health (NIH) Guidelines on Use of Animals in Research. Animal protocols were approved by the National Institute of Dental and Craniofacial Research (NIDCR) Animal Care and Use Committee (ACUC) and the NIH Biosafety Committee. The approval identification number is 09-512.

### *In vivo* vector delivery and detection

Prior to SG administration, all vectors were dialyzed for 3 hours (hrs) against saline. Eight (early treatment) or ten (late treatment) week old mice were anesthetized intramuscularly (im) with a combination of ketamine (100 mg/mL, 1 mL/kg body weight; Fort Dodge Animal Health, Fort Dodge, IA) and xylazine (20 mg/mL, 0.7 mL/kg body weight; Phoenix Scientific, St. Joseph, MO). To each submandibular gland 50 µl vector solution, containing 1×10^11^ vector (ICAM-1/Fc or control vector LacZ) particles plus 1×10^9^ luciferase reporter vector, was administered by retrograde ductal instillation using a thin cannula (Intermedic PE10, Clay Adams, Parsippany, NJ), as previously described [7]. This way of administration is known to effectively and stably transduce the epithelial ductal cells in the SG [15]. At the end of the treatment (20 weeks of age), localization of vector delivery was confirmed by luciferase activity after ip injection of 100 µl (40 mg/ml) luciferin (Gold Biotechnology, St. Louis, MO) and luminescence was imaged by an *in vivo* luminescence imager (Xenogen IVIS® imager using living® image software, Alameda, CA). Vector DNA delivery was also confirmed by homogenization of SG and total genomic DNA was isolated using DNeasy blood & tissue kit (Qiagen, Venlo, the Netherlands). The vector was detected using on an ABI StepOnePlus Real-Time PCR system (Applied Biosystems, Carlsbad, CA) using the following primers specific for the therapeutic gene region: forward 5′-TAGAATTCGCAATGGCTTCA-′3, reverse 5′-CTTCTCTGGGATGGATGGAT and probe 5′-CACCAGGCCCAGGGATCACA-3′.

### Saliva collection and sacrifice

Mice were anesthetized as described above and saliva secretion was induced by subcutaneous (sc) injection of pilocarpine (0.5 mg/kg BW; Sigma-Aldrich, St. Louis, MO). Stimulated whole saliva was collected for 20 minutes from the oral cavity with a hematocrit tube (Drummond Scientific Company, Broomall, PA) placed into a preweighed 0.5 ml microcentrifuge tube, and the volume was determined gravimetrically. At time of sacrifice, serum and the SGs were collected to analyze the effect of treatment compared with the controls.

### Histological assessment and immunohistochemistry

At the end of the study the mice were euthanized and one cross sectional part of the SG was embedded in paraffin and sections were cut at 5 µm. Three non-consecutive sections were stained with hematoxylin and eosin (H&E). The FS was determined for each mouse, in which one focus is defined as an aggregate of 50 or more lymphocytes and the FS defined as the average foci per 4 mm^2^ SG tissue. Slides were scored blindly by at least 2 different researchers. Other paraffin sections were stained with rat anti-mouse B220 (kindly provided by Dr. K. van Gisbergen, University of Amsterdam) after heat-induced citrate antigen-retrieval. Another cross-sectional part of the SG was collected and frozen into OCT compound (Tissue-Tek, Sakura, Zoeterwoude, the Netherlands) and sections were cut at 5 µm. Sections were stained with rat anti-mouse CD4 (eBioscience, San Diego, CA), rat anti-mouse CD8 (eBioscience), rat anti-mouse CD68 (Abcam, Cambridge, MA), and hamster anti-mouse CD11c (Abcam), each at a 1∶100 dilution. Anti-Foxp3 (eBioscience) was used at a 1∶50 dilution using an extra amplification step with biotin labeled tyramide (PerkinElmer, Waltham, MA) and streptavidin labeled HRP (PerkinElmer). Foxp3 is considered a reasonable marker for the presence of Tregs in tissue.

For endogenous ICAM-1 expression, salivary glands of 8, 12, 16 and 20 week old non-treated were frozen in OCT compound, sections were cut at 7 µm and were stained with rat anti-mouse ICAM-1 (Abcam). As secondary antibodies goat anti-rat-HRP (Southern Biotechnology, Birmingham, AL) and goat anti-hamster-HRP (Jackson Immunoresearch, Suffolk, UK) were used. Staining was developed with AEC substrate (Dako, Glostrup, Denmark). Images of the high-power fields taken of the foci were analyzed using the Qwin analysis system (Leica, Cambridge, UK), as described previously [25]. Within each group of treated and control mice, absolute staining intensity values were divided by the average of controls and expressed as relative IOD. In addition, the percentage of total SG tissue surface area taken up by foci was quantified for each section.

### Detection of IgA, IgG and IgM in serum and SGs

Serum was collected by heart puncture just after sacrifice, blood was left to clot for 3 hrs on ice and was spun down for 25 minutes at 2500 g at 4°C. SG homogenates were made by homogenizing a part of the frozen SG in HEPES lysis buffer containing protease inhibitors (Protease Mini, Roche, Indianapolis, IN) overnight at 4°C and total protein was determined with BCA™ protein assay kit (Pierce, Rockford, IL). IgA, IgG and IgM in serum and tissue homogenates were determined using commercially available ELISA kits (Bethyl Laboratories, Montgomery, TX) and final concentrations were corrected for total protein concentration.

### Statistical analysis

Differences between experimental groups in focus scores were assessed using Student's t-tests. Differences in all other experiments were assessed using the non-parametric Wilcoxon's ranksum test or the parametric Student's t-test depending on the Gaussian distribution. All the analyses were performed with GraphPad Prism statistical software (GraphPad Software Inc. version 5.01, La Jolla, CA). A P value≤0.05 was considered to be statistically significant.
